# CSN6 promotes the cell migration of breast cancer cells by positively regulating Snail1 stability

**DOI:** 10.7150/ijms.50206

**Published:** 2020-10-01

**Authors:** Jie Mou, Lulu Wei, Jia Liang, Wenqi Du, Dongsheng Pei

**Affiliations:** 1 Department of Pathology, Xuzhou Medical University, Xuzhou, China.; 2 Jiangsu Key Laboratory of New drug and Clinical Pharmacy, Xuzhou Medical University, Xuzhou, China.; 3 School of Pharmacy, Xuzhou Medical University, Xuzhou, China.; 4 Center for Translational Medicine and Jiangsu Key Laboratory of Molecular Medicine, Medical School of Nanjing University, Nanjing, China.; 5 Department of Human Anatomy, Xuzhou Medical University, Xuzhou, China.

**Keywords:** CSN6, Snail1, cell migration, breast cancer

## Abstract

**Background:** CSN6, a subunit of the highly conserved constitutive photomorphogenesis 9 (COP9) signalosome (CSN), has been reported to be implicated in tumor progression in various kinds of malignant tumors. However, the mechanism underlying CSN6 in the tumor development of breast cancer has not yet been fully elucidated.

**Methods:** CSN6 staining in breast cancer tissues and paracancerous tissues was measured by tissue microarray (TMA) technology. The metastatic effect of CSN6 was measured by cell migration assay. Co-immunoprecipitation study was used to show the interaction between the protein CSN6 and Snail1. Ubiquitination assay was performed to validate whether ubiquitination is involved in the upregulation of Snail1 by CSN6. The impact of CSN6 on tumor metastasis *in vivo* was analyzed using xenotransplantation experiments in BALB/c mice.

**Results:** Here, we demonstrated that CSN6 expression was dramatically increased in breast cancer tissues compared with paired adjacent cancerous tissues. CSN6 promoted the cell migration and wound healing abilities in breast cancer cell lines. Also we showed that CSN6 associates with Snail1 and enhances Snail1 protein level by inhibiting the ubiquitin-mediated degradation of Snail1. Thus, CSN6 is involved in positively regulating the stability of Snail1. We further proved that CSN6 protein level was positively correlated with the Snail1 expression in xenograft model.

**Conclusion:** These findings provide new insight into applicability of using the CSN6-Snail1 axis as a potential therapeutic target in breast cancer.

## Introduction

Breast cancer remains a public health issue on a global level and its survival rate falls from 90% for localized to 20% for metastatic disease 1, 2. Nowadays, the identification of effective interventions to avoid breast cancer is still challenging 3. Stromal invasion and metastasis to distant organs or regional lymph nodes are the characteristics of fully developed breast cancer 4. In addition, obvious metastasis frequently indicates an incurable and chronic disease. The amount of biomolecular markers and targeted therapeutic drugs for breast cancer management has rapidly increased in the past decades. Therefore, further identifying reliable biomarkers and exploring novel targets, is urgently needed for the long-term survival of patients with breast cancer.

The highly conserved constitutive photomorphogenesis 9 (COP9) signalosome (CSN) is an evolutionarily conserved multisubunit protein complex that resembles the 19S lid of the 26S proteasome that is common to all eukaryotes and found in plants and animals 5-7. The COP9 signalosome has been postulated to be associated with protein degradation because of its sequence homology with the 'lid' complex of the 26S proteasome in mammals 8. CSN6, a critical subunit of COP9 signalosome, is involved in ubiquitin-mediated degradation of important proteins implicated in cell cycle progression and signal transduction that relies on the Mprl/Pad1 N-terminal domain 9-13. Furthermore, CSN6 can also coordinate with E3 ligase to mediate ubiquitination and degradation of cancer associated proteins 14. Previous studies have identified that CSN6 was implicated in tumor progression, because it is overexpressed in multiple common types of cancers, such as cervical cancer, thyroid cancer, colorectal cancer, breast cancer, lung cancer, hepatocellular carcinoma and glioblastoma 15-19. In particular, the expression profiles of CSN6 in human cancer patient database from Gene Expression Omnibus and Oncomine illustrate that CSN6 is overexpressed in >50% of cases of glioblastoma, pancreatic cancer and breast cancer 20. However, the molecular mechanism for CSN6 in breast cancer remains obscure.

The epithelial-mesenchymal transition (EMT) is a highly conserved biological process that shifts epithelial cells to a mesenchymal phenotype initially in embryonic development 21. It is well accepted that EMT is critical for the progression of tumor occurrence, migration and metastasis 2. Snail1, a key transcription factor of EMT, was identified in Drosophila, controls large-scale cell movement during neural crest delamination and mesoderm formation 22. Snail1 overexpression was detected in both epithelial and endothelial cells of invasive cancer 23. In addition, Snail1 is related with tumor progression and poor outcome in patients in human malignancies 21, 24. In breast cancer, Snail1 correlates with the tumour grade, poor clinical parameters, a high rate of recurrence, distant metastasis and nodal metastasis for invasive ductal carcinoma 25-28. Furthermore, Snail1 also brings resistance to apoptosis and produces breast cancer stem cell (CSC)-like characteristics by blocking Snail1 ubiquitination 29-31. These suggest that Snail1 is a possible target for therapeutic intervention. However, a comprehensive account of the mechanisms of Snail1 stabilization in breast cancer remains unclear.

In the current study, we utilized unbiased approaches to characterize the mechanism of CSN6 overexpression during the breast cancer tumorigenesis. Furthermore, we identified that CSN6 enhancement leads to Snail1 stabilization and promotes metastasis of breast cancer cells through inhibiting Snail1 ubiquitination. Our findings provided a thoughtful understanding of novel CSN6-Snail1 signaling in promoting breast cancer metastasis.

## Materials and Methods

### Patients and samples

The tissue microarray (TMA) was consisted of 52 cases breast cancer tissues and paired paracancerous tissues, which were collected from the Affiliated Hospital of Xuzhou Medical University. All the patients underwent mastectomy and postoperative adjuvant therapy at Affiliated Hospital of Xuzhou Medical University from March 2012 to May 2016. Detailed clinical information of specimen was recorded accurately, such as clinicopathological parameters including age, TNM stage, lymph node metastasis and others like birth place, marriage and surgery date.

### Ethics statement

Patient tissue samples were obtained with informed consent, under the protocol approved by the ethical review board of the Affiliated Hospital of Xuzhou Medical University. Animal experiments were performed in strict accordance with the protocols approved by the Institutional Animal Care and Use Committee of Xuzhou Medical University.

### Immunohistochemistry and evaluation

After being baked at 65 °C for 2h, TMA slides were dewaxed with dimethylbenzene and then rehydrated with graded alcohol and distilled water. For retrieving antigen, TMA slides were performed in a microwave oven heated at 95 °C with 10 mM citrate buffer (pH 6.0). Endogenous peroxidases were quenched by 3% H2O2 for 20 min. The slides were treated with normal goat serum to inhibit nonspecific staining, then incubated with anti-CSN6 (1:50 dilution, Santa Cruz Biotechnology, USA) overnight at 4 °C and then were incubated with a secondary antibody for 1 h. Diaminobenzidine (DAB; Zhongshan biotech, Beijing, China) was used to produce a brown precipitate. After hematoxylin counterstain and dehydration were completed, the slides were sealed. The immunoreactivity was assessed blindly by two independent observers by microscopy (ZEISS AXIO Scope. A1). The expression of CSN6 was graded as positive when ≥5% of tumor cells showed immunopositivity. Biopsies with <5% tumor cells showing immunostaining were considered negative.

### Cell lines and cell culture

Human normal breast epithelial cell line MCF10A and human breast cancer cell lines BT549, T47D and MDA-MB-231 were obtained from the Shanghai Institute of Biochemistry and Cell Biology, Chinese Academy of Science (Shanghai China). MCF10A cells were cultured in DMEM/F12 Medium, BT549 were cultured in RPMI-1640 Medium, T47D were cultured in DMEM Medium while MDA-MB-231 were cultured in L15 Medium supplemented with 10% fetal bovine serum, 100 U/ml penicillin, 100 μg/ml streptomycin, and incubated in a 37 °C humidified incubator with 5% CO_2_.

### Stable cell line generation

The stable CSN6 overexpressed MDA-MB-231 cells were established by infected with lentiviruses, in which CSN6-control expression vectors and CSN6-overexpression vectors were respectively packed by Gene-Pharma (Soochow, China). MDA-MB-231 cells were infected with lentivirus for 48 h, and then were selected with 2 ng/ml puromycin for 2 weeks, with the medium refreshed every 3 days.

### Small interfering RNA and transient transfections

Small interfering RNA (siRNA) specific for CSN6 (siCSN6) and siRNA NC were purchased from Gene-Pharma (Soochow, China) and transfected by siLentFect Lipid Reagent (Bio-Rad, Hercules, CA, USA) according to the manufacturer's protocol when breast cancer cells were grown to 50%~60% confluency. Six hours after transfection, the medium containing transfection reagents were removed and incubated in fresh medium. The siRNAs sequences were described as follows: si-CSN6 sense: 5'-CCGUGGAAGAGAAGAUUAUTT-3', siCtrl sense: 5'-UUCUCCGAACGUGUCACGUTT-3'.

### Western blot analysis

After digestion and centrifugation of the cells, protein concentration was determined by bicinchoninic acid kit (BCA, Beyotime Biotechnology, Beijing, China). 100 μg proteins were applied onto 12.5% SDS polyacrylamide gel (SDS-PAGE) for electrophoresis and then transferred onto nitrocellulose membrane, and probed with the primary antibodies (CSN6, 1: 2000, Enzo Life Sciences; Snail1, 1: 1500, Abcam; β-actin, 1: 2000, Zhong-shan biotech). After incubating overnight at 4 °C with the primary antibody, membranes were washed with TBS/0.05% Tween-20 (TBST) and incubated with a secondary antibody (1:10000, VICMED) for 2 hours at room temperature. The membranes were then washed and scanned on the chemiluminescence imaging analysis system (Tanon, Biotechnology, Shanghai, China).

### Reverse transcription-polymerase chain reaction (RT-PCR)

Total RNA was extracted with Trizol Reagent (Thermo Fisher). The initial reaction uses the reverse transcriptase enzyme to generate DNA from RNA. Firstly the reaction was set up on ice in a 0.2 ml thin-walled PCR tube. Incubate samples in a thermocycler for 1 h at 37-42 °C. And the reverse-transcribed single-stranded DNA was denatured at 95 °C for 2 min and place on ice. Then pour a 1% agarose gel with ethidium bromide in 1×TAE. Run 5-10 ml of the PCR reaction along with a DNA ladder at 100 V for 30-45 min. Finally the gel was visualized using a UV gel documentation system.

### Co-immunoprecipitation

Cell lysates for immunoprecipitation were incubated on a rocker with indicated antibody (CSN6, 1: 50, Enzo Life Sciences) at 4 °C overnight for co-immunoprecipitation (co-IP) assay. The lysis buffer contained a cocktail of protease/phosphatase inhibitors (sigma) and cell lysates were immunoprecipitated by Protein A/G beads (Santa Cruz). Then the beads were centrifuged at a low speed for 10 min and supernatant was discarded. Dried beads were mixed with 1× loading buffer and boiled for 5 min. Lysate samples were loaded onto gels following western blot analysis.

### Migration assay

Transwell chambers with a pore size of 8 μm were applied to examine cell migration. 1 × 10^4^ breast cancer cells were seeded in serum-free medium in the upper chamber. The medium containing 10% FBS was added to the lower chamber. After cultured for 24 h in a 37 °C incubator, the cells were fixed in methanol and stained with leucocrystal violet. Cells in upper chamber were removed and the number of cells which traversed the membrane was determined by counting the leucocrystal violet stained cells. Stained cells were counted under a microscope in the whole field.

### Wound healing assay

The transfected BT-549 and MDA-MB-231 cells were seeded in 6-well plates. When cell culture reached about 95% confluence, the cell monolayer was slowly scratched with a sterile 200μl pipette tip. We washed the well with fresh medium to remove cellular debris and cultured cells again. The wound was imaged at 0 and 24 h. All assays were repeated three times.

### Ubiquitination assay

BT-549 and MDA-MB-231 cells were transfected with indicated plasmids. After 24 hours, cells were treated with 50 μg/mL of MG132 (Selleck Biotechnology, USA) for 6 h. The ubiquitinated proteins were immunoprecipitated with anti-Snail1. The protein complexes were then resolved by SDS-polyacrylamide gel and probed with anti-ubiquitin to visualize the level of ubiquitination.

### Xenograft mouse metastatic model

Twenty female 6-week-old BALB/c nude mice were purchased from Beijing Huafukang Bioscience (Beijing, China). Stable MDA-MB-231 cells (LV-CSN6^OE^ and LV-NC) were concentrated to 2 × 10^6^/100 μl PBS and injected into mice via the lateral tail vein. After 30 days, all mice were euthanized and lungs were then excised and photographed. The number of metastatic tumors per lung were counted and recorded. Then, the lung tissues were harvested, embedded, fixed, and prepared for H&E, IHC staining and western blot.

### Statistical analysis

Data were analyzed using SPSS 16.0 software. Statistical significance of Student's *t*-test was presented for two-group comparisons. More than two groups were examined by one-way or two-way analysis of variance. The correlations between CSN6 expression and clinicopathologic characteristics were analyzed using Pearson's chi-square test or Fisher's exact test in breast cancer tissues and para-cancer tissues. Differences between groups were statistically significant when *P* < 0.05.

## Results

### CSN6 is increased in breast cancer tissues and is associated with clinicopathologic parameters in breast cancer

**Figure 1:**
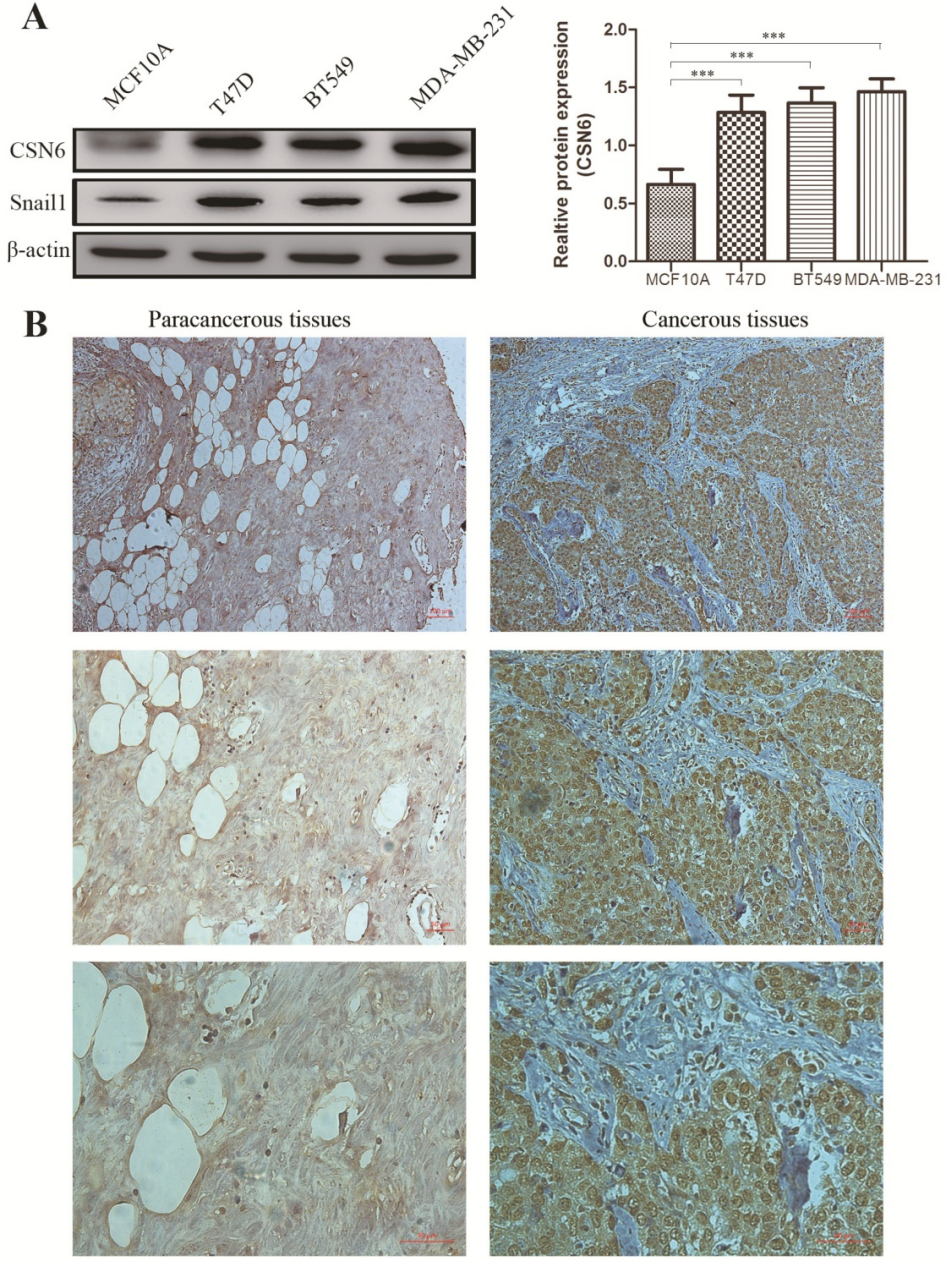
** CSN6 expression is increased in human breast cancer cells and tissues.** (A) Western blot analysis was used to evaluate the CSN6 and Snail1 expression in various breast cancer cell lines and normal breast epithelial cell line MCF10A. The data represent means ± S.D. ***P < 0.001. (B) Representative photos of CSN6 expression patterns in breast cancer tissues. The right side shows the breast cancerous tissues, whereas the left side depicts matched paracancerous tissues (original magnification, 100×). The magnifying detail of the immunohistochemical analysis for each case can be shown on the middle panel (original magnification, 200×) and bottom panel (original magnification, 400×).

To investigate whether the expression of CSN6 is changed in breast cancer cells, western blot analysis was performed to examine the CSN6 expression in normal breast epithelial cell line MCF10A and three human breast cancer cell lines. Results showed that the expression of CSN6 in breast cancer cells is higher than that in normal breast epithelial cells (Figure 1A). To evaluate the correlation between CSN6 expression and clinicopathological parameters (according to TNM classification), we collected breast cancer tissues and corresponding adjacent tissues from 52 breast cancer patients. Then CSN6 expression was analyzed using IHC in the TMA of 52 breast cancer specimens to investigate the clinical implication of CSN6 protein. We found that CSN6 expression is localized in the cytoplasm. In addition, the expression of CSN6 was higher in cancerous tissues than in paracancerous tissues (Figure 1B). In paracancerous tissues, positive CSN6 staining was recorded in 23.1% (12 of 52 cases). Of the 52 patients with breast cancer tissues, positive expression of CSN6 was observed in 59.6% (31 of 52 cases) (Table 1). Because TNM stage is an important prognostic marker for breast cancer patients, so we investigated if CSN6 expression correlates with TNM stage. Our data demonstrated that CSN6 showed a significant correlation with histological grade (*P*< 0.05, Table 1). Meanwhile, statistical analysis revealed that increased CSN6 expression significantly correlated with depth of invasion pT (*P*< 0.05, Table 1) and lymph node metastasis pN (*P*< 0.05, Table 1).

### CSN6 promotes breast cancer cells migration *in vitro*

**Figure 2:**
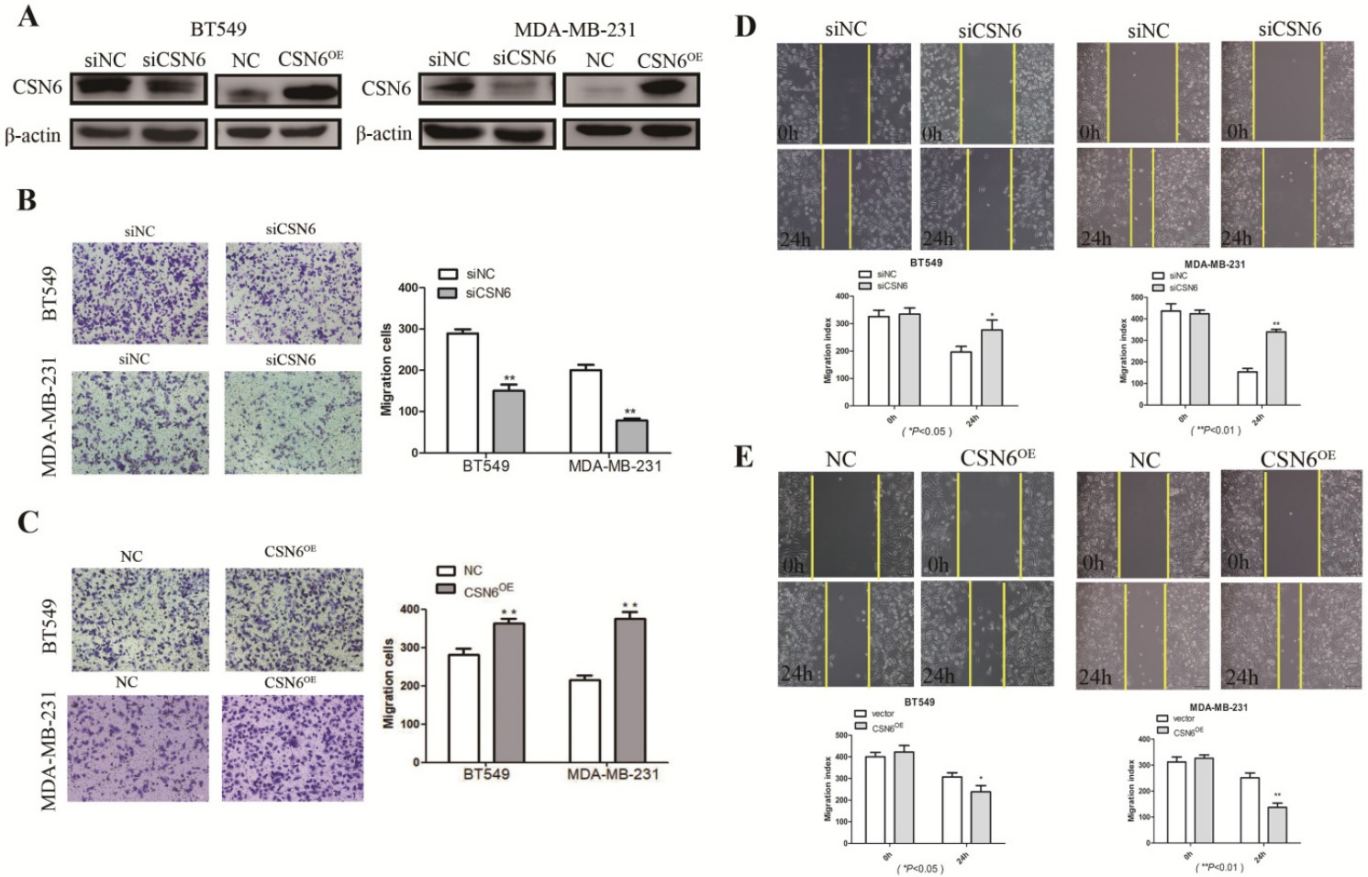
** CSN6 promotes migration and invasion in breast cancer cells.** (A) Western blot was used to detect the expression efficiency of CSN6 when CSN6 gene vector or Si-CSN6 was transfected into two kinds of breast cancer cells. (B-C) Cell migration assays were performed in BT-549 and MDA-MB-231 cells after CSN6 knockdown or expression, respectively. (D-E) The breast cancer cells motilities were measured through testing the wound closure after CSN6 overexpression and knockdown in BT-549 and MDA-MB-231 cells, respectively. The data represent means ± S.D. *P < 0.05, **P < 0.01, ***P < 0.001.

It is acknowledged that malignant cells are characterized by possessing the capabilities of adhesion and migration. Hence, we investigated the role of CSN6 in motility of breast cancer cells. To access the effect of CSN6 on cell migration ability, western blot was performed and showed that siCSN6 and CSN6 overexpression plasmid were successfully transfected into breast cancer cells (Figure 2A). Cell migration was measured by transwell assays, and we found that CSN6 knockdown BT549 and MDA-MB-231 cells could harbor weaker abilities to penetrate through the inserts (Figure 2B). On the contrary, overexpression of CSN6 increased the number of cells passing through the inserts (Figure 2C). Wound healing assays showed that knockdown of CSN6 decreased the capacity for BT549 and MDA-MB-231 cell motility (Figure 2D). Furthermore, a significant increase was revealed in the wound closure of CSN6 overexpression cells when compared with the control cells (Figure 2E). Overall, the results indicated that CSN6 promoted the migration in breast cells *in vitro*.

### CSN6 stabilizes Snail1 by impeding Snail1 ubiquitination

**Figure 3:**
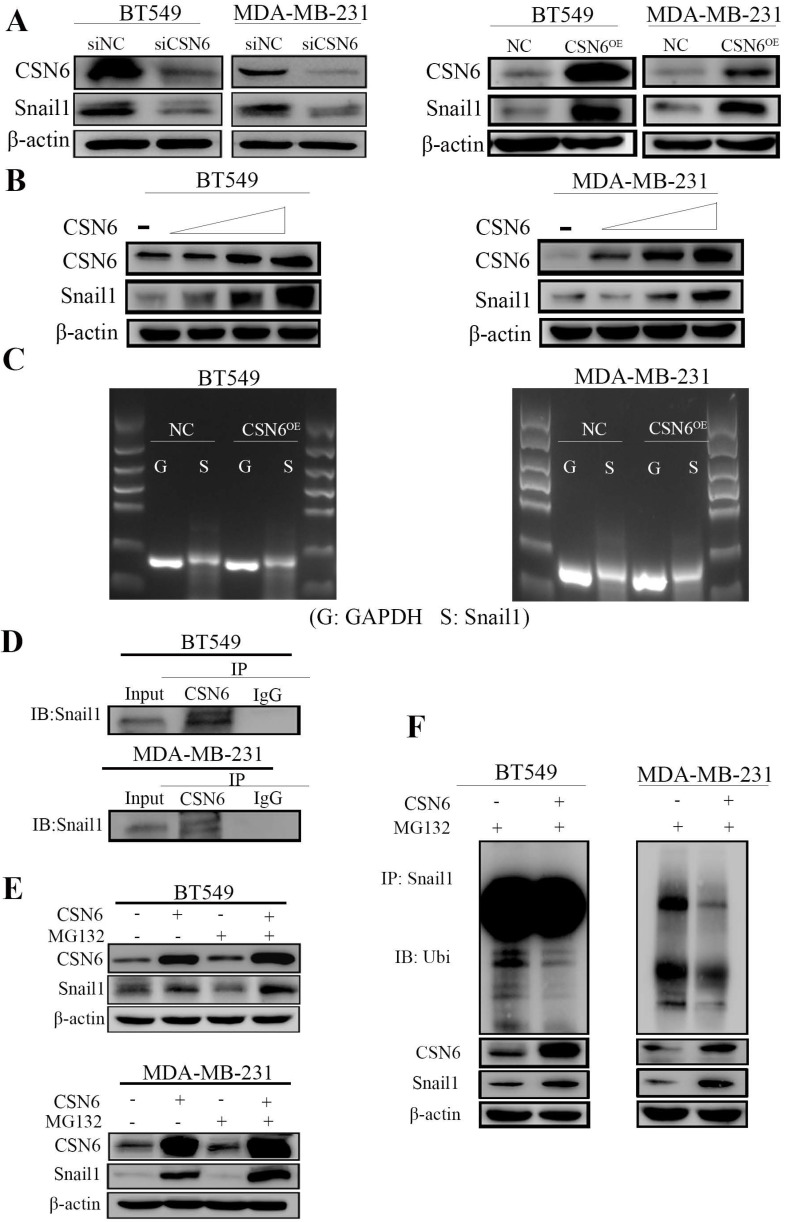
** CSN6 positively regulates the expression of Snail1.** (A) Western blot analysis of Snail1 expression after CSN6 overexpression or knockdown in BT-549 and MDA-MB-231 cells. (B) CSN6 enhanced the protein level of Snail1 in a dose-dependent (2 µg, 4 µg, 6 µg) manner in breast cancer cell lines. (C) The mRNA levels of Snail1 were checked when BT-549 and MDA-MB-231 cells were transfected with plasmids expressing CSN6 or pcDNA3.1. (D) The breast cancer cells lysates were analyzed by immunoprecipitation with CSN6 and were immunoblotted with anti-Snail1. CSN6 interacted with endogenous Snail1. (E) CSN6 prevents Snail1 degradation via proteasomal degradation. BT-549 and MDA-MB-231 cells were transfected with CSN6-expression vectors and treated with or without MG132 for 6 h, then subjected to Western blot analysis using an anti-Snail1 or anti-actin antibody. (F) BT-549 and MDA-MB-231 cells were transfected with control vector or CSN6. The cell lysates were immunoprecipitated with anti-Snail1 and immunoblotted with anti-ubiquitin antibody.

It is identified that Snail1 is the main regulator of EMT and EMT is the process of cancer cells invasion and migration. In addition, Snail1 is a zinc finger transcription factor, and plays an important role in tumorigenesis through silencing the expression of E-cadherin and inducing EMT. To investigate the mechanism by which CSN6 regulates tumor metastasis in breast cancer cells, Western Blot was performed to examine the relationship between CSN6 and Snail1 protein levels in BT549 and MDA-MB-231 cells. In Figure 3A, data showed that the protein level of Snail1 decreased after CSN6 knockdown and the expression level of Snail1 protein increased after CSN6 overexpression in the breast cancer cells. Meanwhile, the expression of Snail1 in breast cancer cells is higher than that in normal breast epithelial cells which is consistent with CSN6 expression (Figure 1A). Furthermore, CSN6 could positively regulate the stability of Snail1 in a dose-dependent manner (Figure 3B). The above results prompted us to study how CSN6 up-regulates Snail1 protein. We found that the mRNA level of Snail1 did not change after CSN6 overexpression in breast cancer cell lines (Figure 3C). This suggested that Snail1 expression may be regulated by CSN6 at or after translation level. The results of co-immunoprecipitation showed that CSN6 and Snail1 proteins bound to each other (Figure 3D). We next investigated how CSN6 interacted with Snail1 to regulate the protein level of Snail1. Breast cancer cells were transfected with the indicated plasmids and treated with the proteasomal inhibitor MG132. Results showed that Snail1 protein levels increased in the presence of both CSN6 and MG132 (Figure 3E). Then we performed ubiquitination assay and found that CSN6 overexpression decreased the ubiquitination level of Snail1 (Figure 3F). Taken together, these results indicated that CSN6 promotes Snail1 stability through reducing ubiquitination.

### CSN6 promotes tumor metastasis via up-regulating snail1 in xenograft model

**Figure 4:**
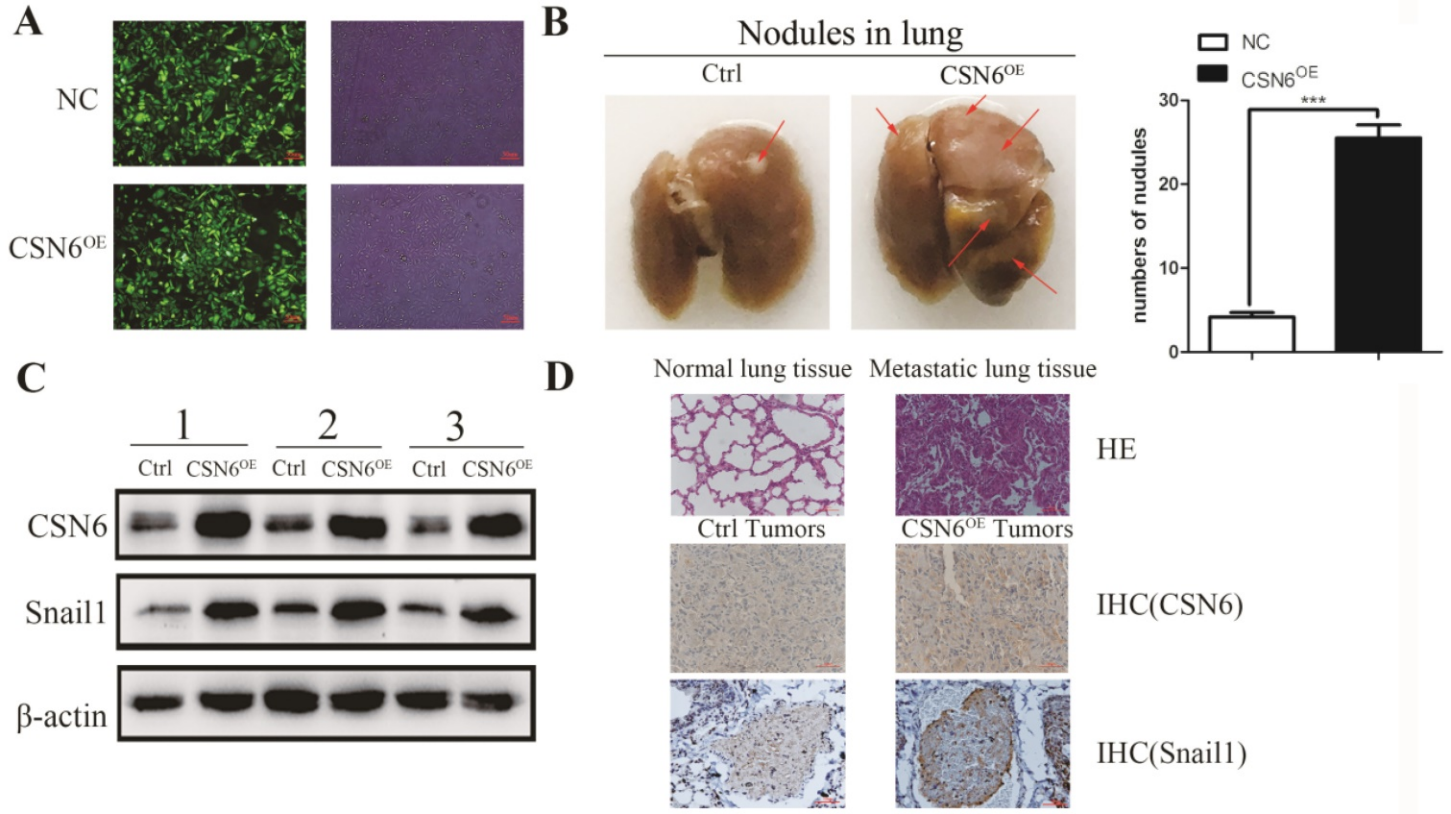
** CSN6 promotes breast cancer cells metastasis *in vivo*.** (A) The photograph of MDA-MB-231 cells stably transfected CSN6 under a fluorescence microscope. (B) Numbers of lung metastasis nodules from each mouse were counted. (C) Western blot detected the protein expressions of CSN6 and Snail1 in metastasis tumors. (D) H&E staining of lung sections. Representative images of IHC for CSN6 and Snail1 staining metastasis tumors (Original magnifications, 400×).The data represent means ± S.D. ***P < 0.001.

To identify the role of CSN6 in the metastatic potency of breast cancer cells *in vivo*, lentiviruses packed with CSN6-NC or CSN6 overexpression vectors were transfected into MDA-MB-231 cell lines, and the fluorescence intensity was detected by Olympus light microscope (Figure 4A). CSN6 overexpression cells were injected into tail veins of the nude mice to establish a tumor metastasis model and compared numbers of lung nodules with those among control nude mice injected with CSN6-NC cells. After 30 days, the nude mice were sacrificed and the lung tissue was dissected for observation. We found that the number of metastatic lung nodules in CSN6-overexpression mice were significantly higher than the control group (Figure 4B). To detect the CSN6 expression and snail1 protein levels *in vivo*, western blot analysis was performed to examine the extracted protein of metastasis tumor. The results indicated that the CSN6 and snail1 expression levels were up-regulated in CSN6 overexpression group versus control group (Figure 4C). Furthermore, the lung tissues were paraffin embedded for subsequent analysis and the expression of CSN6, Snail1 was detected via Immunohistochemistry staining (Figure 4D). The results demonstrated that CSN6 promoted tumor metastasis *in vivo* and up-regulated the protein level of Snail1, which was consistent with the results mentioned *in vitro* experiments.

## Discussion

Accumulating evidence has suggested that CSN plays an important role in the regulation of multiple cancers and could be an effective target for therapeutic intervention. In addition, CSN is involved in regulating cullin-based E3 ligases and could potentially be altered in tumors 32, 33. Previous studies have shown that CSN3 and CSN5 could accelerate the cancer cell proliferation and growth 34, 35. CSN6, as a critical subunit of CSN, is upregulated in numerous malignances 20, 36, 37. It is identified that CSN6 is implicated in modulating the biological functions of cancer progression, including the DNA repair, cell cycle and tumorigenesis 16, 38. Given these vital roles, a better perceptive of the biological functions of CSN6 signaling regulation will provide potential approaches for inhibiting CSN6-induced cancerogenesis. Although few studies documented the roles of CSN6 in tumorigenesis, the mechanism roles of CSN6 in breast cancer tumorigenesis remain to be determined. Our study reveals that CSN6 promotes the migration and metastasis of breast cancer cells, indicating that CSN6 functions as an oncogene in breast cancer cells.

In the last several years, abnormal expression of CSN6 has been revealed to be associated with the clinicopathological characteristics of tumors and the prognosis of several tumor patients. The present study has suggested that CSN6 is not only highly expressed in tumors, but also plays an important role in the biological behavior of tumor cells. It is reported that CSN6 could stabilize the E3 ubiquitin ligase COP1 in human colorectal cancer cell line HCT116 and human embryonic kidney cell line HEK-293T, thereby accelerating the cyclin-dependent protein kinase inhibitor p27 that involved in the G1 phase of cell cycle to regulate cell proliferation 39. In addition, overexpression of CSN6 could also reduce the ubiquitination of the proto-oncoprotein c-Jun mediated by the E3 ubiquitin ligase MEKK1 and promote cell proliferation in the human embryonic kidney cell lines 40. In glioma cells, CSN6 stabilizes epidermal growth factor receptor (EGFR) by enhancing the self-ubiquitination of E3 ubiquitin ligase CHIP, thereby promoting glioma cells proliferation, migration and invasion 17. Meanwhile, CSN6 could bind to the E6 oncoprotein of human papillomavirus (HPV) and inhibit the polyubiquitination of E6 to reduce its degradation, thereby regulating the activity of p53 in cell proliferation and apoptosis to affect the development of cervical cancer 41. These studies indicated that CSN6 was carcinogenic in the occurrence and development of cancer. In the current study, we investigated the role of the CSN6 in breast cancer and found that the expression of CSN6 in breast cancer cell lines was higher than that of normal breast epithelial cell lines. We also collected 52 pairs of breast cancer tissues and corresponding para-cancerous tissues for immunohistochemistry analysis. The results revealed that the expression of CSN6 in cancer tissues was higher than that of para-cancerous tissues, and it was significantly related to TNM staging. These results suggest that CSN6 plays a critical role in the progress of breast cancer. Subsequently we investigated the biological function of CSN6 in breast cancer.

Although the combined application of surgical treatment, radiotherapy and chemotherapy has greatly improved the five-year survival rate of breast cancer patients, the cancer recurrence still plagues various patients. The recurrent breast cancer frequently has distant metastasis of tumors, making the patient lost the opportunity for surgery. EMT is an important biological process in which malignant tumor cells derived from epithelial cells and acquire the capabilities of migration and invasion 42, 43. During EMT, the expression of epithelial marker gene E-cadherin decreases and the expression of interstitial marker genes increases, including Snail, N-cadherin and vimentin 42. Snail, an important zinc finger transcription factor that drives the EMT process, includes three members Snail1 (Snail), Snail2 (Slug) and Snail3 (Smuc). They bind to the E-box DNA sequence through the carboxyl terminal zinc finger domain to inhibit the expression of epithelial marker genes 44. Studies have demonstrated that the activity of Snail1 is enhanced in the vicinity of the promoter of the epithelial marker E-cadherin, thereby inhibiting the expression of E-cadherin and promoting the progress of EMT 45. In addition to inhibiting the expression of epithelial marker genes, Snail1 could also activate gene expression of interstitial phenotypes, such as fibronectin, N-cadherin, and matrix metalloproteinase family 46. This study we investigated the roles of CSN6 in breast cancer cells by specifically interfering or overexpressing CSN6. CSN6 overexpression conferred on cells tumorigenic properties and contributed to migration of breast cancer cells *in vitro* and* in vivo*, CSN6 knockdown resulted in a reverse trend. Western Blot analysis showed that CSN6 could up-regulate the expression of Snail1 protein in a dose-dependent manner, and CO-IP analysis showed that CSN6 could bind with Snail1. These results indicate that CSN6 affects the EMT process of cancer cells by up-regulating the expression of Snail1 protein and promotes cell migration.

Next, we investigated how CSN6 specifically regulates the protein level of Snail1. Through RT-PCR analysis we found that the mRNA level of Snail1 did not change significantly after CSN6 overexpression in breast cancer cell lines. Thus, we speculated that CSN6 induced up-regulation of Snail may be regulated at the post-transcriptional level. It is identified that the CSN plays an important role in protein degradation because it has a high degree of homology with the “lid” complex of the 26S proteasome in mammals 8. Previous studies have shown that CSN6 play an important role in the regulation of proteasome activity depending on the N-terminal domain of Mprl/Pad1 10-12. Our study showed that CSN6 prevented Snail1 degradation via proteasomal degradation. In many cases, modulation of protein levels largely relies on ubiquitination-mediated degradation by the 26S proteasome. Increasing evidence illustrates that the deubiquitination activity of CSN5 is critical for cancer progression 47. Previous studies have shown that CSN5 led to the metastasis and EMT activation of cancer cells through decreasing ZEB1 ubiquitination 48. We then validated whether ubiquitination is involved in CSN6-mediated Snail1 regulation. Ubiquitination assay was performed and revealed that CSN6 overexpression decreased the ubiquitination level of Snail1. The above results suggest that CSN6 promotes Snail1 stability via impeding ubiquitination of Snail1. On the basis of our data, it is possible that CSN6-mediated Snail1 stabilization may be a common feature of various types of cancer. Our discovery of CSN6 in stabilizing Snail1 updates the paradigm with evidence that CSN has a versatile role in modulating various types of E3 ligases. CSN6 probably functions to inhibit Snail1 ubiquitination and then switches the E3 ligase target from Snail1 itself to other proteins. Such a hypothesis is worth further investigation.

In summary, we draw noteworthy conclusions that CSN6 had a positive impact on Snail1 stability, and then contributing to the enhanced metastasis potential of breast cancer cells. Furthermore, CSN6 is associated with clinicopathologic parameters in breast cancer and it could act as valid prognostic factor of breast cancer. CSN6 induces EMT and enhances metastasis of breast cancer cells by reducing Snail1 ubiquitination. Therefore, CSN6 might be considered as a promising novel therapeutic target for breast cancer.

## Figures and Tables

**Table 1:** Clinical characteristics of 52 breast cancer patients and CSN6 expression

Pathology Character	N	CSN6 staining		*P* Value
		Negative	Positive	
**Tissue**				0.0001***
Cancerous	52	21	31	
Paracancerous	52	40	12	
**Age (year)**				0.508
≤55	27	15	12	
>55	25	13	12	
**TNM Stage**				0.016*
I	9	8	1	
II	30	12	18	
III	13	4	9	
**pT status**				0.024*
pT1	11	9	2	
pT2	29	10	19	
pT3	7	3	4	
pT4	5	0	5	
**pN status**				0.01*
pN0	34	22	12	
pN+	18	3	15	
